# Propofol protects against high glucose-mediated endothelial injury via inhibition of COX2 and iNOS expressions: Propofol protects against endothelial injury

**DOI:** 10.3724/abbs.2022020

**Published:** 2022-03-03

**Authors:** Jiayun Shao, Juan Ding, Lihong Lu, Wenting Hou, Fei Wang, Zhirong Sun, Hui Jiang, Yanjun Zhao

**Affiliations:** Department of Anesthesiology Fudan University Shanghai Cancer Center; Department of Oncology Shanghai Medical College Fudan University Shanghai 200032 China

**Keywords:** propofol, high glucose, COX2, iNOS, SP1

## Abstract

Perioperative hyperglycemia is a common metabolic disorder in the clinic. Hyperglycemia, via upregulation of E74-like ETS transcription factor 3 (ELF3), induces cyclooxygenase 2 (COX2) and inducible nitric oxide synthase (iNOS) expressions, thus leading to endothelial apoptosis and vascular endothelial injury. Propofol is a widely used anesthetic. In the present study, we explored whether and how propofol protects against high glucose-induced COX2 and iNOS expressions in human umbilical vein endothelial cells (HUVECs). We found that high glucose level decreases cell viability and increases COX2 and iNOS expressions in HUVECs. Our data also indicated that ELF3 overexpression participates in high glucose-mediated cell viability reduction and high glucose-induced COX2 and iNOS expressions. Moreover, propofol treatment improves high glucose-mediated reduction in cell viability and decreases COX2 and iNOS expressions via inhibition of ELF3 expressions. Furthermore, specificity protein 1 (SP1) was found to regulate ELF3 expression, thus mediating endothelial injury. Propofol inhibits high glucose-induced SP1 expression. High glucose increases the abundance of SP1 bound to the ELF3 promoter, which can be reversed by propofol treatment. The protective effect of propofol is reversed by SP1 overexpression. In conclusion, propofol downregulates high glucose-induced SP1 expression, thus attenuating high glucose-induced ELF3 expression, inhibiting high glucose-induced COX2 and iNOS expressions, and improving high glucose-mediated cell viability reduction in HUVECs.

## Introduction

Perioperative hyperglycemia is a common clinical metabolic disorder in nondiabetic [1] and diabetic patients [2]. Perioperative hyperglycemia often occurs because of physiological stress and excessive glucose transfusion during the perioperative period. Hyperglycemia-mediated endothelial injury participates in the pathogenesis of cardiovascular complications [ 3– 5]. It has been reported that inflammatory enzymes, such as cyclooxygenase 2 (COX2) and inducible nitric oxide synthase (iNOS), are involved in hyperglycemia-induced endothelial injury and the occurrence of hyperglycemia-related cardiovascular complications [ 6, 7].

E74-like ETS transcription factor 3 (ELF3) was originally identified as an epithelial restricted ETS factor [8]. Inflammatory stimulation up-regulates ELF3 expression in vascular endothelial cells [9]. Moreover, ELF3 is associated with the upregulation of COX2 [10] and iNOS [9] expression by modulating transcription, thus participating in endothelial injury. Specificity protein 1 (SP1) has been reported to modulate ELF3 expression and influence hyperglycemia-induced COX2 and iNOS expressions in endothelial cells [11].

Propofol (2,6-diisopropylphenol) is a commonly used intravenous anesthetic clinically. Previous studies have indicated that propofol prevents high glucose-induced endothelial oxidation and injury [ 12– 14]. However, whether propofol attenuates high glucose-induced COX2 and iNOS expression is not known.

In the present study, we explored whether and how propofol protects HUVECs against high glucose-induced COX2 and iNOS expressions in human umbilical vein endothelial cells (HUVECs).

## Materials and Methods

### Cell culture and reagents

HUVECs (ATCC, Manassas, USA) were incubated in Dulbecco’s modified Eagle medium (DMEM; HyClone Laboratories, Logan, USA) with 5 mM glucose and 10% fetal bovine serum in an incubator containing 5% CO _2_ at 37°C. The cells were subcultured when they reached 90% confluence. The fourth passage of HUVECs was used in the present study.

Propofol (Sigma, St Louis, USA) was dissolved in dimethyl sulfoxide (DMSO; Sigma). The final concentration of DMSO was adjusted to 0.01% in each solution to avoid nonspecific effects.

### Cell transfection

The Flag-tagged coding sequence of human ELF3 and SP1 were cloned into the lentiviral vector pPCDH-CMV-MCS-EF1-puro (Biotend, Shanghai, China) to construct ELF3 and SP1 overexpression plasmids. The plasmids were transfected into HUVECs with the use of Lipofectamine 3000 (Invitrogen, Waltham, USA) according to the manufacturer’s instructions. HUVECs were also transfected with siRNA against ELF3 and SP1 using Lipofectamine 3000 (Invitrogen) according to the manufacturer’s instructions.

The sequences of si-ELF3 (Biotend) were as follows: si-ELF3a, sense, 5′-GCCAUUGACUUCUCACGAUdTdT-3′, anti-sense, 5′-AUCGUGAGAAGUCAAUGGCdTdT-3′ and si-ELF3b, sense, 5′-GCCAUGAGGUACUACUACAdTdT-3′, anti-sense, 5′-UGUAGUAGUACCUCAUGGCdTdT-3′. The sequences of si-SP1 (Biotend) were as follows: si-SP1a, sense, 5′- CAUCCAAGGCUGUGGGAAAdTdT-3’, anti-sense, 5′-UUUCCCACAGCCUUGGAUGdTdT-3′ and si-SP1b, sense, 5′-GCACAAACGUACACACACAdTdT-3′, anti-sense, 5′-UGUGUGUGUACGUUUGUGCdTdT-3′.

### Study design

HUVECs were incubated in 25 mM glucose DMEM for 3 days. During the last 2 h of incubation, the cells were treated with different concentrations (0.2, 1, 5, and 25 μM) of propofol. The optimal concentration of propofol with maximal protective effects against 25 mM glucose-mediated cell viability reduction was determined. These treatment conditions were employed in the subsequent experiments in which the HUVECs were incubated and divided into three groups as follows: Group 1: the cells were incubated in 5 mM glucose as a control; Group 2: the cells were incubated in 25 mM glucose for 3 days; and Group 3: the cells were incubated in 25 mM glucose for 3 days and cocultured with 5 μM propofol for the last 2 h.

### Cell viability assay

Cell Count Kit-8 (CCK-8) kit (Dojindo Molecular Technologies, Tokyo, Japan) was used to assess cell viability. The cells were plated in 96-well plates at a density of 5×10 ^3^ cells/well. After different treatments, 10 μL of CCK-8 solution was then added to each well, and the cells were further incubated for 2 h. The optical density (OD) values were measured at 450 nm with a microplate reader and they were used as an indicator of the relative cell viability.

### Western blot analysis

Whole-cell protein extracts were produced with cell lysis buffer (Cell Signaling Technology, Danvers, USA). The same amount of protein (60 μg) obtained from different groups of HUVECs was separated by 8% or 10% SDS-PAGE and transferred to PVDF membranes. After being blocked in 5% fat-free milk solution, the membranes were incubated with the specific primary antibody at 4°C overnight. The primary antibodies used were anti-β-actin, anti-SP1, anti-ELF3, anti-COX2 and anti-iNOS monoclonal antibodies (1:1000; Proteintech, Wuhan, China). After that, the membranes were washed with Tris-buffered saline containing Tween 20, and the membranes were incubated with the corresponding secondary antibody (1:1000; Proteintech) for 1 h at room temperature. Subsequently, the membranes were washed, and the specific protein bands were detected using the ECL system ( Millipore Corporation, Billeruca, USA). The respective densities of the protein bands were analyzed by Scan-gel-it software (UNSCAN-IT gel 6.0; Silk Scientific Inc., Orem, USA). In this study, β-actin was used as the loading control for the whole cell extract.

### Quantitative PCR

Total RNA was extracted using Trizol reagent (Tiangen Biotech, Beijing, China). cDNA was synthesized using Hifair® II 1st Strand cDNA Synthesis Super-Mix for qPCR (gDNA digester plus; Yeasen, Shanghai, China). qPCR was carried out by using the Hieff UNICON® qPCR TaqMan Probe Master Mix (Yeasen) to analyze the gene expressions of β-actin, SP1, ELF3, iNOS, and COX2 with the QuantStudio 7 Flex Real-Time PCR System (Applied Biosystems, Waltham, USA). The qPCR primer sequences used in the present study are listed in Table 1.

**Table:** **Table 1** Sequences of primers used in this study

Gene	Primer sequence (5′→3′)
Actin	Forward: ATGCCCTGAGGCTCTTTTCCAGCC Reverse: CCAGGATGGAGCCACCGATCCACA
SP1	Forward: CGAAGTAGCAGCACAGGCAGTAG Reverse: GGAGCGGCAGCCACAACATAC
ELF3	Forward: TGGAAGTGACGTGGACCTGGATC Reverse: GACGCCTTCATGCCGATTCTCC
iNOS	Forward: ACTACAGGCTCGTGCAGGACTC Reverse: CCACCACTCGCTCCAGGATACC
COX2	Forward: CCATTGACCAGAGCAGGCAGATG Reverse: TGGCTTCCAGTAGGCAGGAGAAC

### Chromatin immunoprecipitation (ChIP) assay

ChIP assays were carried out using a Simple Chromatin immunoprecipitation (ChIP) Plus Sonication Chromatin IP kit (Cell Signaling Technology, Danvers, USA). Briefly, 1% formaldehyde was used to fix the cells (1×10 ^7^) at room temperature for 10 min. A Microson XL ultrasonic cell disruptor XL (Misonix, Farmingdale, USA) was used to shear the chromatin. After that, 10 μL of the solution was used as the input. The rest of the solution was incubated with anti-SP1 antibody (Cell Signaling Technology) or a negative control IgG at 4°C for 12 h. After purification, the enriched DNA sequences were detected by PCR. The ELF3 oligonucleotide primers were as follows: forward, 5′-GTGTACAACACACCTGCATA-3′, and reverse, 5’-GAATGCATGGTTATTCCCAT-3′.

### Immunofluorescence microscopy

The cells were seeded onto glass slides with the corresponding treatment. After fixation with 4% paraformaldehyde, the cells were permeabilized with 0.3% Triton X-100 for 5 min. Then, HUVECs were blocked with 1% bovine serum albumin at room temperature for 1 h, followed by incubation with anti-SP1 antibodies at 4°C for 12 h. DAPI was used to stain the nuclei. The images were acquired with a confocal Leica fluorescence microscope (Wetzlar, Germany).

### Statistical analysis

The sample sizes were determined by assessing the high glucose-mediated cell viability reduction in the pilot experiments. We anticipated that statistical significance could be achieved with a sample size of 5 in the in vitro experiments. Data are expressed as the mean±SD. Statistical comparisons were performed with one-way analysis of variance followed by Bonferroni-corrected pairwise comparisons. A value of *P*<0.05 was considered statistically significant.

## Results

### Effect of propofol on high glucose-induced endothelial injury and COX2 and iNOS expressions in HUVECs

**Figure 1:**
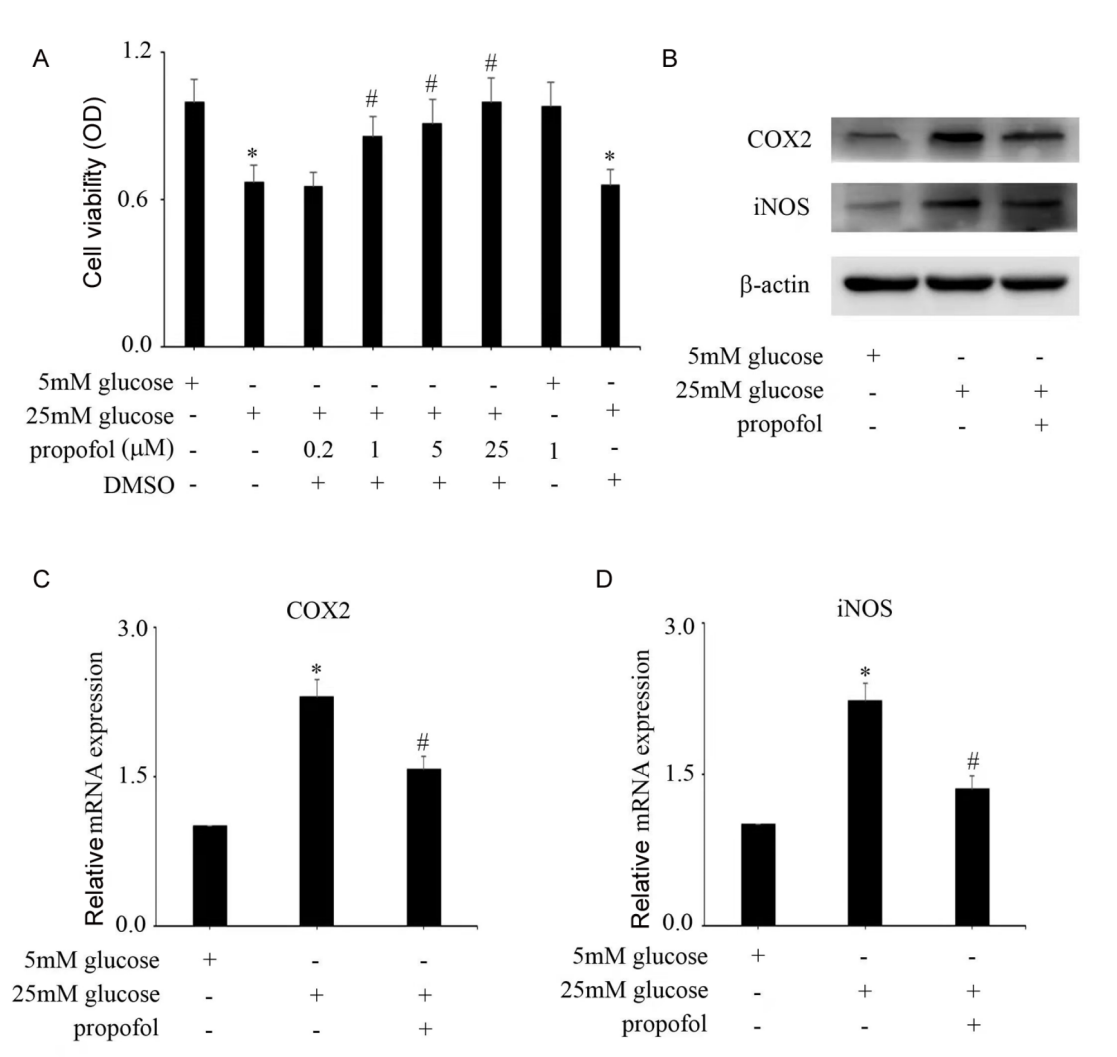
Figure 1 Effect of propofol on high glucose-induced endothelial injury and COX2 and iNOS expressions in HUVECs (A) CCK8 was used to detect cell viability in HUVECs with the corresponding treatments. (B) Western blot analysis of COX2 and iNOS expressions in HUVECs with the corresponding treatments. (C) The mRNA expression of COX2 was examined by qPCR in HUVECs with the corresponding treatments. (D) The mRNA expression of iNOS was examined by qPCR in HUVECs with the corresponding treatments. *P<0.05, compared with the control group; #P<0.05, compared with high glucose treatment.

High glucose-mediated COX2 and iNOS upregulation leads to vascular endothelial injury [ 6, 7]. Propofol has been reported to improve high glucose-mediated endothelial injury in HUVECs. Therefore, we hypothesized that propofol may improve high glucose-mediated endothelial injury via inhibition of COX2 and iNOS expressions. Compared with 5 mM glucose treatment, high glucose treatment induced a marked decrease in HUVEC viability. Propofol improved the high glucose-mediated reduction in cell viability in a concentration-dependent manner in HUVECs ( Figure 1A). Incubation of HUVECs with 1 μM propofol for 2 h caused a marked reversal of cell reduction under high glucose conditions ( Figure 1A). Meanwhile, the solvent DMSO, at the concentration used in the experiments, had no effect on cell viability ( Figure 1A). This method was used in the following experiments to study the potential signaling pathways by which propofol improves high glucose-mediated endothelial cell injury.

Compared with 5 mM glucose treatment, high glucose increased COX2 and iNOS protein ( Figure 1B) and mRNA ( Figure 1C,D) expressions. Moreover, propofol treatment inhibited high glucose-induced COX2 and iNOS protein ( Figure 1B) and mRNA ( Figure 1C,D) expressions.

### The protective effect of propofol against high glucose-mediated endothelial injury is achieved via inhibition of ELF3 expression in HUVECs

**Figure 2:**
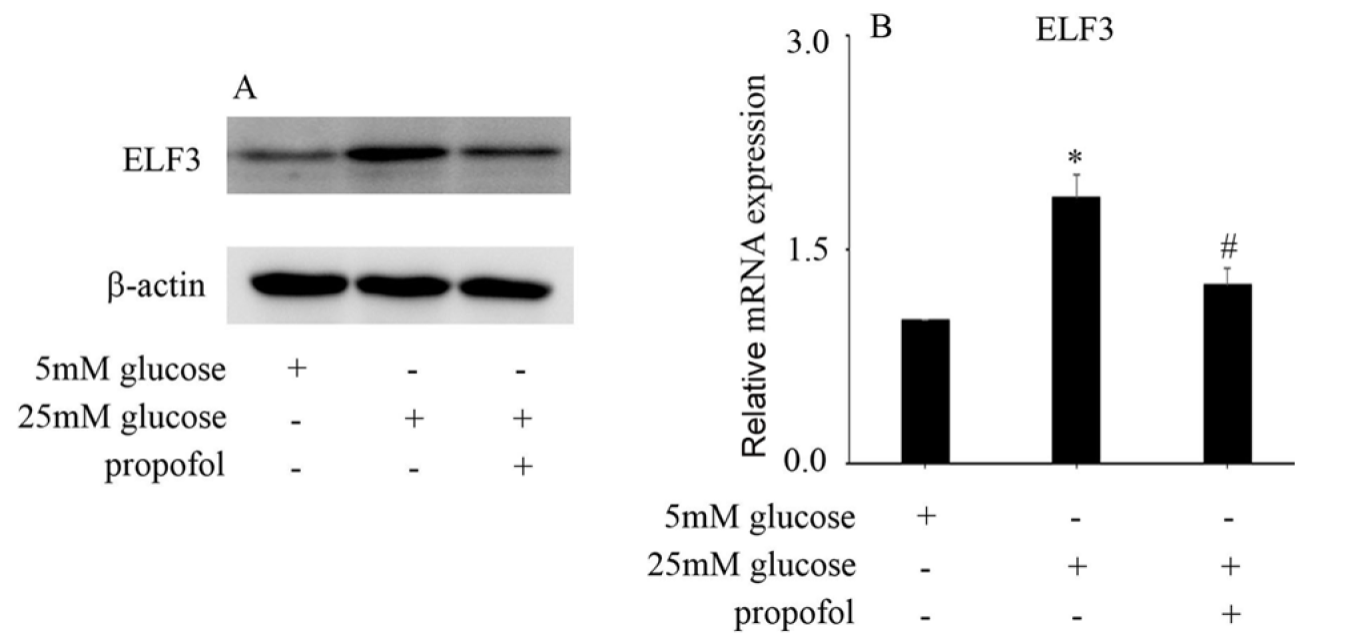
Figure 2 Effect of propofol on high glucose-induced ELF3 expression in HUVECs (A) Western blot analysis of ELF3 expression in HUVECs with the corresponding treatments. (B) The mRNA expression of ELF3 was examined by qPCR in HUVECs with the corresponding treatments. *P<0.05, compared with the control group; #P<0.05, compared with high glucose treatment.

It has been reported that ELF3 regulates the expressions of iNOS [9] and COX2 [10]. Therefore, we determined whether propofol inhibits COX2 and iNOS expressions via inhibition of ELF3 in hyperglycemic HUVECs. Our data indicated that high glucose increased ELF3 protein ( Figure 2A) and mRNA ( Figure 2B) expressions in HUVECs. Moreover, propofol was found to inhibit ELF3 expression in hyperglycemic HUVECs ( Figure 2).

**Figure 3:**
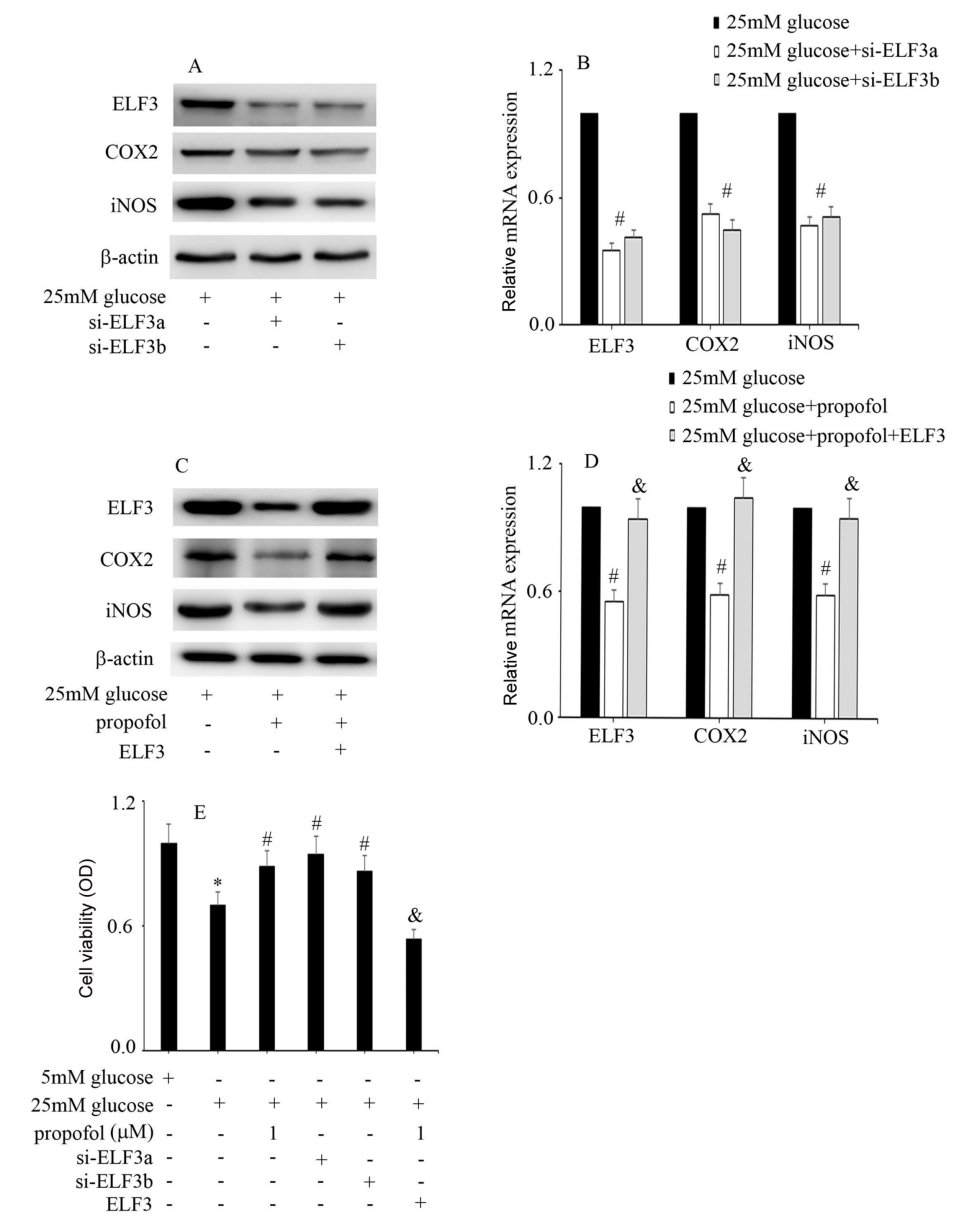
Figure 3 The protective effect of propofol against high glucose-mediated endothelial injury is achieved via inhibition of ELF3 expression in HUVECs (A) Western blot analysis of ELF3, COX2 and iNOS expressions in HUVECs with the corresponding treatments. (B) The mRNA expressions of ELF3, COX2 and iNOS were examined by qPCR in HUVECs with the corresponding treatments. (C) Western blot analysis of ELF3, COX2 and iNOS expression in HUVECs with the corresponding treatments. (D) The mRNA expressions of ELF3, COX2 and iNOS were examined by qPCR in HUVECs with the corresponding treatments. (E) CCK-8 assays were used to detect the cell viability in HUVECs with the corresponding treatments. *P<0.05, compared with the control group; #P<0.05, compared with high glucose treatment; and &P<0.05, compared with high glucose+propofol treatment.

Next, we explored whether propofol protects against high glucose-induced endothelial injury and COX2 and iNOS expressions via inhibition of ELF3 expression. Our data indicated that high glucose-induced COX2 and iNOS expressions were inhibited by si-ELF3 ( Figure 3A,B), which was similar to propofol treatment. Moreover, the protective effect of propofol against high glucose-induced COX2 and iNOS expressions was inhibited by ELF3 overexpression ( Figure 3C,D). Consistently, the high glucose-mediated reduction in cell viability was improved by si-ELF3, which is similar to propofol treatment ( Figure 3E). Furthermore, the protective effect of propofol against high glucose-mediated cell viability reduction was reversed by ELF3 overexpression ( Figure 3E). These data indicated that the protective effect of propofol against high glucose-mediated endothelial injury is achieved via inhibition of ELF3 expression in HUVECs.

### Effect of propofol on high glucose-induced SP1 expression in HUVECs

**Figure 4:**
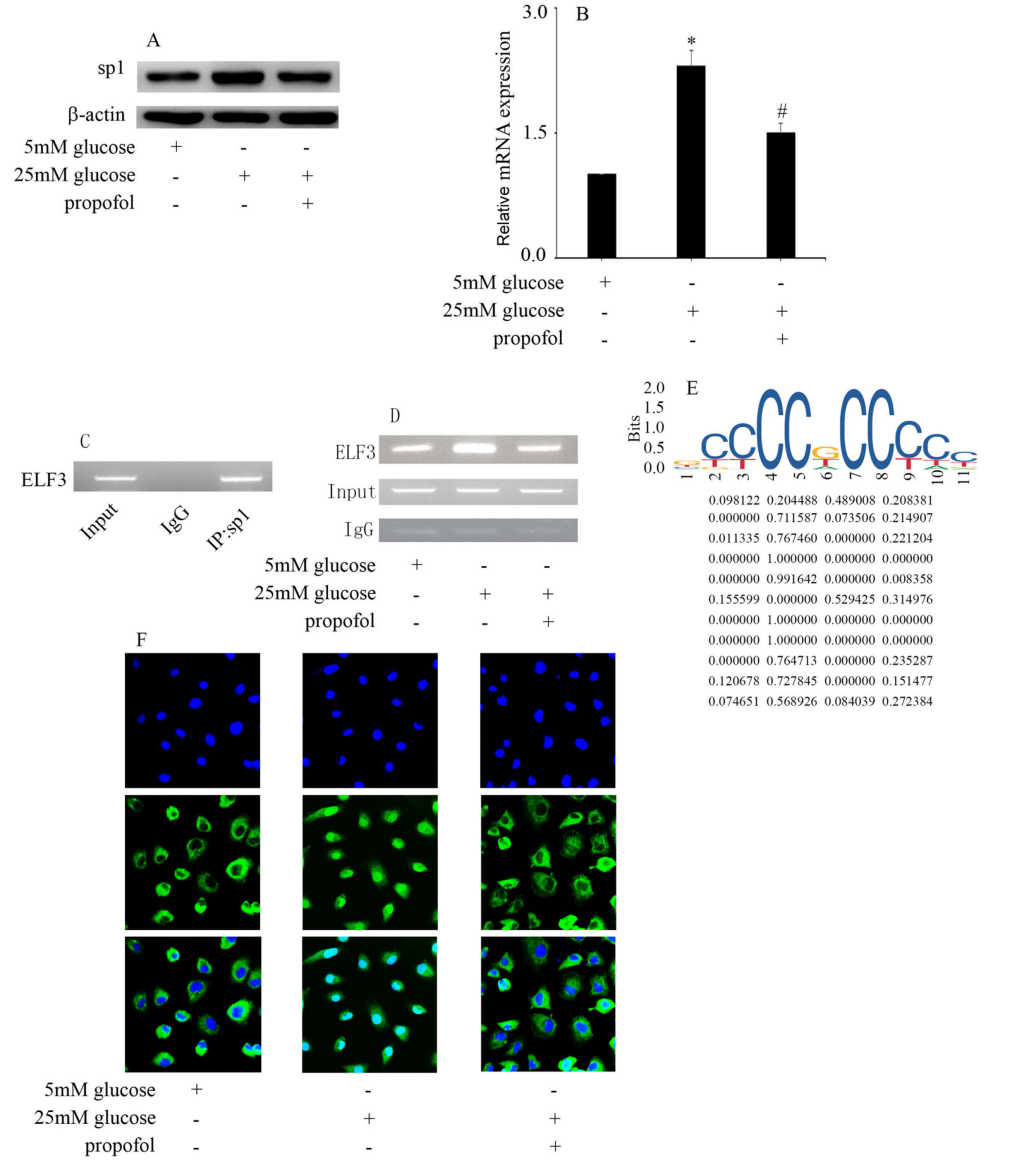
Figure 4 Effect of propofol on high glucose-induced SP1 expression in HUVECs (A) Western blot analysis of SP1 expression in HUVECs with the corresponding treatments. (B) The mRNA expression of SP1 was examined by qPCR in HUVECs with the corresponding treatments. (C) SP1 was enriched at the ELF3 promoter region. (D) High glucose increased the abundance of SP1, which binds with the ELF3 promoter, and this effect was reversed by propofol treatment. (E) The putative SP1 binding site in ELF3. The motif logo and position weight matrix are shown in the upper and lower panels, respectively. (F) High glucose treatment induced SP1 nuclear translocation in HUVECs, which was counteracted by propofol treatment. *P<0.05, compared with the control group; #P<0.05, compared with high glucose treatment.

It has been reported that SP1 is involved in regulating the expression of ELF3 in hyperglycemic endothelial cells [11]. Therefore, we determined whether propofol inhibited ELF3 expression via inhibition of SP1 in hyperglycemic HUVECs. Our data showed that high glucose increased SP1 protein ( Figure 4A) and mRNA ( Figure 4B) expressions in HUVECs. Moreover, propofol was found to inhibit SP1 expression in hyperglycemic HUVECs ( Figure 4A,B). Furthermore, high glucose increased the abundance of SP1, which bound to the *ELF3* promoter, and this effect was reversed by propofol treatment ( Figure 4C,D). The potential SP1 binding site is shown in Figure 4E. Furthermore, high glucose treatment induced SP1 nuclear translocation in HUVECs, which was counteracted by propofol treatment ( Figure 4F).

### The protective effect of propofol against high glucose-induced endothelial injury and ELF3 expression is achieved via a decrease of SP1 expression in HUVECs

**Figure 5:**
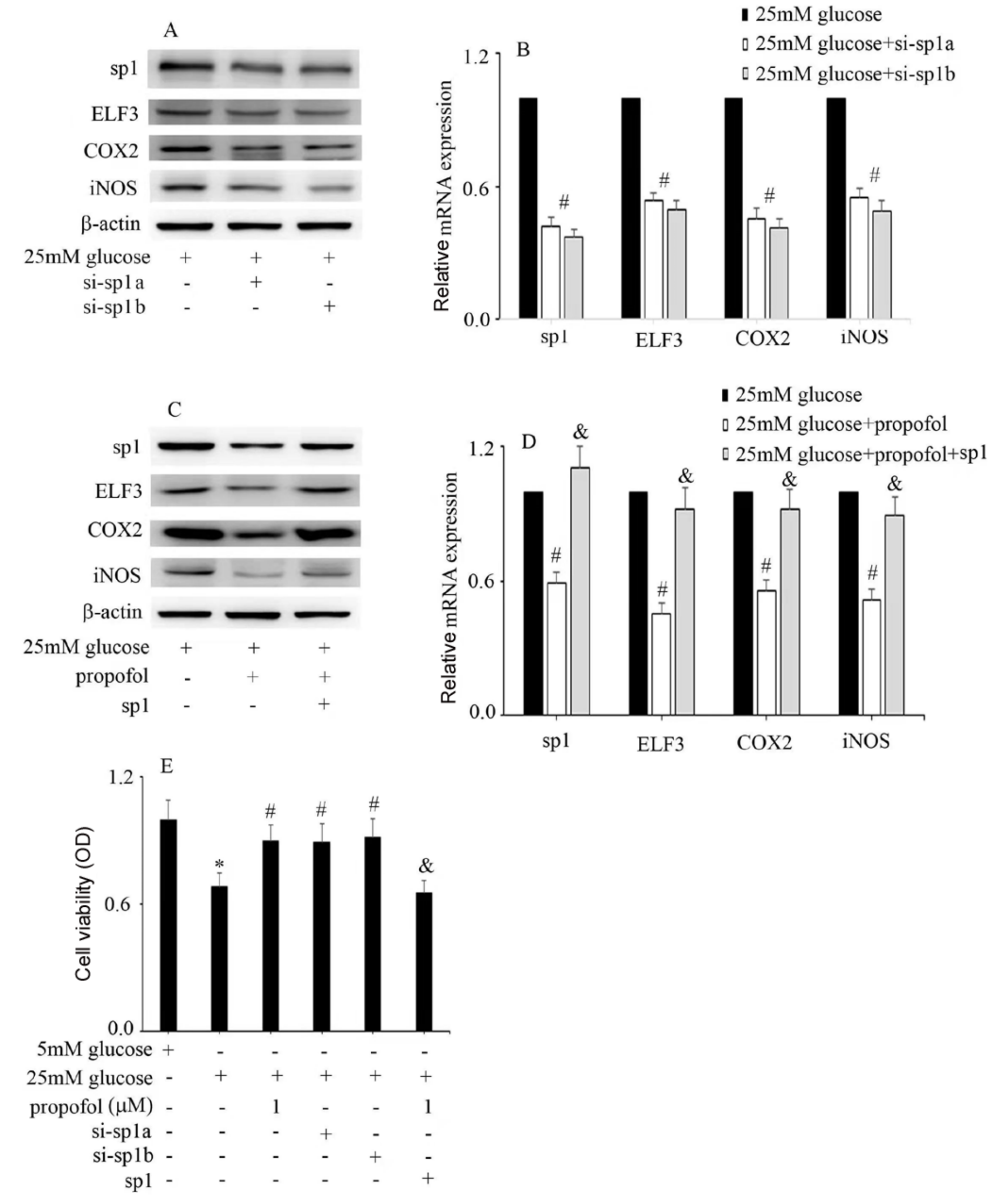
Figure 5 The protective effect of propofol against high glucose-induced endothelial injury and ELF3 expression is achieved via decreasing SP1 expression in HUVECs (A) Western blot analysis of SP1, ELF3, COX2 and iNOS expressions in HUVECs with the corresponding treatments. (B) The mRNA expressions of SP1, ELF3, COX2 and iNOS were examined by qPCR in HUVECs with the corresponding treatments. #P<0.05, compared with the high glucose group. (C) Western blot analysis of SP1, ELF3, COX2 and iNOS expressions in HUVECs with the corresponding treatments. (D) The mRNA expressions of SP1, ELF3, COX2 and iNOS were examined by qPCR in HUVECs with the corresponding treatments. #P<0.05 compared with the high glucose group and &P<0.05, compared with the high glucose + propofol treatment group. (E) CCK-8 assay was used to detect cell viability in HUVECs with the corresponding treatments. *P<0.05, compared with the control group; #P<0.05, compared with high glucose treatment; and &P<0.05, compared with high glucose+propofol treatment.

**Figure 6:**
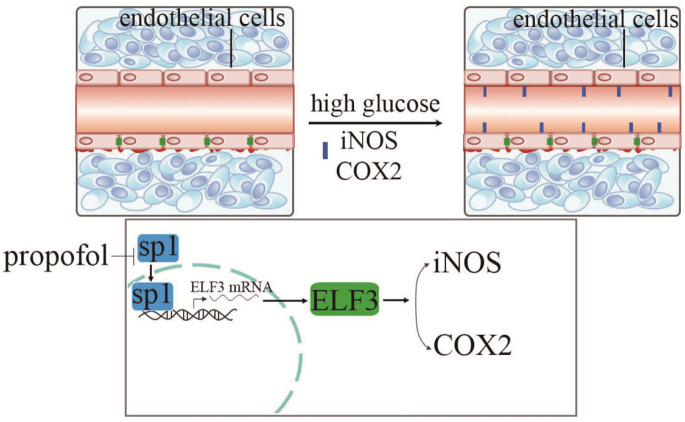
Figure 6 Schematic representation of the working model High glucose, via upregulation of SP1 expression, activates ELF3 transcription, thus inducing iNOS and COX 2 expression, as well as endothelial injury. The protective effect of propofol against high glucose-mediated endothelial injury is achieved via inhibition of SP1 expression and nuclear translocation in HUVECs

Furthermore, we explored whether propofol protects against high glucose-induced endothelial injury and COX2, iNOS and ELF3 expressions via inhibition of SP1. Our data indicated that high glucose-induced ELF3, COX2 and iNOS expressions were inhibited by si-SP1 ( Figure 5A,B), which was similar to propofol treatment. Moreover, the protective effect of propofol against high glucose-induced ELF3, COX2 and iNOS expressions was inhibited by SP1 overexpression ( Figure 5C,D). Consistently, the high glucose-mediated reduction in cell viability was improved by si-SP1, which was similar to propofol treatment ( Figure 5E). Furthermore, the protective effect of propofol against high glucose-mediated cell viability reduction was reversed by SP1 overexpression ( Figure 5E). These data indicated that the protective effect of propofol against high glucose-mediated endothelial injury is achieved via inhibition of SP1 expression and nuclear translocation in HUVECs ( Figure 6).

## Discussion

In the present study, we found that propofol improved high glucose-mediated reduction in cell viability, and that the protective effect of propofol may be achieved by down-regulating SP1 expression and inhibiting ELF3-mediated COX2 and iNOS expressions in hyperglycemic HUVECs.

Hyperglycemia plays an important role in vascular endothelial injury [15], and it is involved in the pathogenesis of cardiovascular complications in diabetic patients [ 3– 5]. The inflammatory factor COX2 has been reported to participate in high glucose-induced endothelial injury and hyperglycemia-related complications [6], including diabetic retinopathy [16], vascular leakage [17], and abnormalities [18]. Similarly, iNOS also participates in high glucose-mediated endothelial injury [ 7, 19, 20]. ELF3 has been reported to participate in endothelial inflammatory processes via upregulating the expressions of iNOS [9] and COX2 [10]. Moreover, ELF3 silencing protected against high glucose-induced COX2 and iNOS expression and endothelial injury [11]. Therefore, ELF3 may be a target to improve high glucose-mediated endothelial cell injury.

Previous studies have indicated that SP1 is involved in hyperglycemia-mediated endothelial injury [ 21– 23]. Activation of SP1 transcription augments high glucose-mediated plasminogen activator inhibitor-1 gene expression [ 21, 22]. Under high glucose conditions, SP1 activates keap1 transcription, resulting in endothelial oxidation and diabetic nephropathy [23]. Moreover, SP1 activation induces COX2 and iNOS expressions via upregulation of ELF3 expression [11]. These results are similar to those in our study and indicate that SP1 may be a potential target for hyperglycemia-induced endothelial injury [ 9– 11].

Propofol is a common intravenous anesthetic. In addition to its sedative and hypnotic effects, other effects of propofol have been investigated in recent years. Propofol has been reported to attenuate high glucose-mediated vascular endothelial oxidation via inhibition of p66shc expression [ 12, 13]. Propofol attenuates high glucose-induced endothelial cell inflammation and apoptosis via inhibition of PKC activity [ 13, 14]. Moreover, propofol improves high glucose-mediated endothelial dysfunction by restoring endothelial nitric oxide synthase uncoupling [20]. Furthermore, propofol attenuates hyperglycemia-mediated cardiac hypertrophy via activator of transcription 3 in rats [24]. In the present study, propofol was found to improve high glucose-mediated cell viability ( Figure 1) and inhibit high glucose-induced COX2 and iNOS expressions ( Figure 1) by attenuating ELF3 expression ( Figure 3). Moreover, we found that propofol reduced high glucose-induced ELF3 expression via inhibition of SP1 expression ( Figure 5). Furthermore, high glucose-mediated reduction in cell viability was improved by si-SP1 ( Figure 5E) and si-ELF3 ( Figure 3E), which is similar to the effect of propofol. The protective effect of propofol on high glucose-mediated cell viability reduction was reversed by SP1 overexpression ( Figure 5E) and ELF3 overexpression ( Figure 3E). These data indicate that the protective effect of propofol against high glucose-mediated endothelial injury is achieved via inhibition of SP1 expression.

Nevertheless, the current study has some limitations. This study was implemented in HUVECs, which is an *in vitro* system. There may be some differences from in vivo settings in consideration of drug effectiveness and toxicity. Therefore, further *in vivo* studies are necessary to confirm the present results. Moreover, the mechanism by which propofol affects SP1 expression was not explored. Further studies are required to clarify whether propofol is a gamma-aminobutyric acid receptor agonist [ 25, 26] and whether it affects SP1 expression by activating GABA.

In summary, in the present study we found that high glucose upregulates ELF3 expression by inducing SP1 expression, thus resulting in COX2 and iNOS overexpressions and endothelial cell viability reduction ( Figure 6). More importantly, this study shows that propofol downregulates high glucose-induced SP1 expression and then attenuates high glucose-induced ELF3 expression, thus inhibiting high glucose-induced COX2 and iNOS expressions and improving high glucose-mediated cell viability reduction in HUVECs.
